# Combining ^18^F‐FDG PET/CT and Serum Lactate Dehydrogenase for Prognostic Evaluation of Small Cell Lung Cancer

**DOI:** 10.3389/fphar.2020.592768

**Published:** 2020-10-28

**Authors:** Xiaoping Lin, Zizheng Xiao, Yingying Hu, Xu Zhang, Wei Fan

**Affiliations:** ^1^ Department of Nuclear Medicine, Sun Yat-Sen University Cancer Center, Guangzhou, China; ^2^ State Key Laboratory of Oncology in South China, Collaborative Innovation Center for Cancer Medicine, Sun Yat-Sen University Cancer Center, Guangzhou, China

**Keywords:** small cell lung cancer, PET/CT, prognosis, maximum standardized uptake value, lactate dehydrogenase, glycolysis

## Abstract

**Objective:** To investigate the value of using ^18^F-FDG PET/CT in combination with serum lactate dehydrogenase (LDH) for prognostic evaluation of newly diagnosed small cell lung cancer (SCLC).

**Methods:** We reviewed 118 patients with pathologically proven SCLC who underwent ^18^F-FDG PET/CT imaging evaluation in our hospital. Among these patients, 64 patients had extensive disease (ED) and 54 patients had limited disease (LD). The maximum standardized uptake value (SUV_max_) of primary tumor was measured. A Cox proportional hazards model was used to evaluate age, sex, performance status, serum LDH, tumor stage and SUV_max_ on the prediction of overall survival (OS) and median survival time (MST) of patients. Subgroup analysis was performed based on the SUV_max_ in combination with serum LDH.

**Results:** According to the Receiver Operating Characteristic (ROC) curve, the optimal cut-off value of SUV_max_ was 10.95. The AUC was 0.535 (95% CI: 0.407–0.663). The patients were divided into four groups according to the SUV_max_ (higher or lower than 10.95) and LDH (higher or lower than 245 U/L). The univariate and multivariate analyses showed that curative thoracic radiotherapy, Prophylactic Cranial Irradiation (PCI) and the combination of primary tumor SUV_max_ ≤ 10.95 and LDH ≤ 245 U/L were prognostic factors of OS in patients with all patients (*p <* 0.05). Smoking status, PCI, the combination of primary tumor SUV_max_ ≤ 10.95 and LDH ≤ 245 U/L were prognostic factors of OS in patients with LD (*p <* 0.05). N stage and PCI were significant predictors in both of univariate and multivariate analysis of OS for ED SCLC (*p <* 0.05). Among all patients, 27 had low SUV_max_ and normal LDH, and their MST was 36 months (95% CI: 12.98–59.02). Ninety-one patients had high SUV_max_ and/or high LDH, and their MST was 20 months (95% CI: 15.47–24.53). The difference between these two groups was significant (*p* = 0.045). In patients with LD, 16 patients had low SUV_max_ and normal LDH, and their MST was 72 months (95% CI: 26.00–118.0). Thirty-eight patients had high SUV_max_ and/or high LDH, and their MST was 27 months (95% CI: 20.80–33.21). The difference between these two groups was significant (*p* = 0.012). In patients with ED SCLC, 10 patients had low SUV_max_ and normal LDH, with an MST of 18 months (95% CI: 13.69–22.32. Fifty-four patients had high SUV_max_ and/or high LDH, and their MST was 12 months (95% CI: 10.61–13.39). The difference of MST between these two groups was not statistically significant (*p* = 0.686).

**Conclusion:** ^18^F-FDG PET/CT in combination with serum LDH were prognostic factors of overall survival in patients with SCLC. The prognosis of patients with LD SCLC who had low SUV_max_ of primary tumor and normal LDH was better than those with high SUV_max_ and/or high LDH.

## Introduction

Small cell lung cancer (SCLC) is the most common neuroendocrine tumor of the lung. It accounts for 10–15% of lung cancers (Travis, 2012; Torre et al., 2015). SCLC has unique biological behaviors, including rapid growth, short doubling time, invasive and distant metastasis in the early stage of the disease (Rodriguez and Lilenbaum, 2010). Although SCLC is sensitive to radiotherapy and chemotherapy, recurrence or disease progression occurs frequently, and the prognosis is poor. In the past few decades, the overall survival of SCLC has not improved. In recent years, targeted therapy and molecular mechanism of pathogenesis were explored in basic and clinical studies. However, the development of treatments for SCLC is slow because of the heterogeneity of tumor. The pathogenesis and the driving genes of SCLC remain unclear (Kalemkerian and Schneider, 2017).

The main prognostic factors for SCLC include tumor staging, the patient’s physical condition, such as physical status, immune function status, etc. and tumor biological characteristics. The principal prognostic factors of SCLC are staging, physical status, weight loss, tumor burden, and elevated serum biomarkers. Staging is the principal consideration for prognosis and treatment strategies. Patients with SCLC are conventionally staged according to a two-stage system as limited disease (LD) and extensive disease (ED). This system was developed by the Veterans Administration Lung Cancer Study Group (VALG) and has been widely used for a long time. LD is defined as disease confined to one hemithorax that can be encompassed in a tolerable radiation field, whereas ED is defined as disease extending outside the thorax or the existence of malignant pleural effusion. The TNM staging is applied in SCLC (Vallières et al., 2009; Carter et al., 2014). Currently, SCLC staging is based on anatomical images, which only reflect the size of the tumor and the range of invasion.

Serum lactate dehydrogenase (LDH) is used as an important prognostic factor in lymphoma, multiple myeloma, lung cancer and nasopharyngeal carcinoma. Serum LDH is elevated in 50–60% of patients with SCLC at diagnosis and is also critical in the prognosis of SCLC (Byhardt et al., 1986; Stahel et al., 1989; Sagman et al., 1991; Quoix et al., 2000; Chen et al., 2018; Hsieh et al., 2018).

With the development of molecular imaging, ^18^F-FDG PET/CT is increasingly used for clinical diagnosis and prognosis (Warburg, 1956; Vander Heiden et al., 2009; Liao et al., 2012; Doherty and Cleverland, 2013; Bamji-Stocke et al., 2018). ^18^F-FDG PET/CT shows the distribution and metabolism of glucose in the whole body and can be used not only to determine the staging and location of tumors but also to confirm tumor proliferation according to the metabolic index. ^18^F-FDG PET/CT can provide metabolic information, tumor biological behavior and characteristics, as well as tumor burden.

Combination of comprehensive ^18^F-FDG PET/CT and serum LDH can not only reflect tumor glucose intake, glycolysis inflow and glycolysis effluent but also show the distribution of tumors in the whole body. It can also reveal tumor biological behavior, tumor burden and other prognostic key information in addition to staging.

## Materials and Methods

### Ethical Approval

This study was approved by the Ethics committee of the Sun Yat-sen University Cancer Center Institutional Review Board and was conducted following the Declaration of Helsinki.

### Participants

A total of 118 patients were recruited in this study. Patients’ age ranges from 30 to 81 years old, mean age 59 years. All patients were pathologically diagnosed with SCLC at Sun Yat-Sen University Cancer Center from June 2005 to December 2016. Patients received no anticancer treatment before enrollment. Whole-body ^18^F-FDG PET/CT was carried out 4 weeks before treatment. Serum LDH assessment was performed 2 weeks before or after ^18^F-FDG PET/CT scan.

The serum LDH before treatment was assayed based on the spectrophotometric absorbance of nicotinamide-adenine dinucleotide at 340 nm after catalytic oxidation of lactate to pyruvate. The normal reference range was 109–245 IU/L.

Images were acquired with two integrated PET/CT devices (Discovery ST, GE and Biograph mCT; Siemens). PET was performed to cover the identical axial field of view after CT scan. PET images were iteratively reconstructed with CT data for correcting attenuation. For quantitative analysis, irregular regions of interest were placed over the most intense area of ^18^F-FDG accumulation. The maximum standardized uptake value (SUV_max_) was calculated using the following formula: maximum pixel value with the decay-corrected region-of-interest activity (MBq/kg)/[injected dose (MBq)/body weight (kg)]. The PET images were evaluated by two experienced physicians of nuclear medicine. SUV_max_ of the primary tumor was automatically measured on the workstation.

The general characteristics of the patients, including KPS score, smoking status, body weight, serum LDH, overall survival time (OS), and median survival time (MST) were recorded.

All statistical analyses were performed using SPSS (version 13.0; SPSS Inc.). Statistical difference between the groups were analyzed using one-way ANOVA and t tests where appropriate. *p* values of less than 0.05 were considered statistically significant. Survival curves were calculated by the method of Kaplan and Meier. The log-rank method was used to test the statistical significance of the differences between survival curves and to perform comparisons adjusted for other prognostic factors. *p* values for difference between proportions were calculated with Fisher’s exact test (two-tailed). Correlations between LDH levels (normal or elevated) and specific metastatic sites were assessed with the product moment correlation coefficient.

## Results

### Patients Characteristics

**FIGURE 1:**
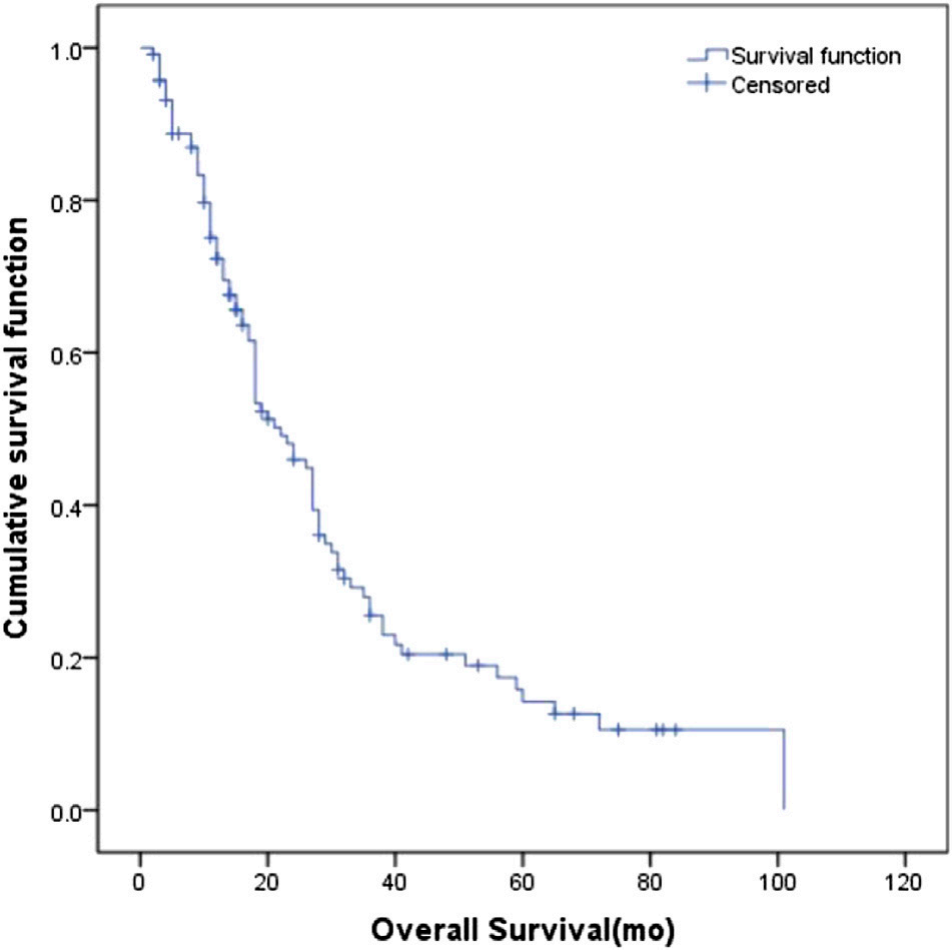
The overall survival curve of the 118 patients with SCLC.

The clinical characteristics of the 118 patients including age, gender, performance status (KPS), tumor stage, body weight, smoking status and treatment management are presented in Table 1. The median age of the patients was 59 years (ranges 30–81 years). At the time of analysis, 32 (27.1%) patients were alive, and the other patients had passed away. The median follow-up time was 18 months (2–101 months). The estimated MST for the entire cohort was 22 months (95% CI: 16.8–27.2 months) (Figure 1), with 1-year, 2-years, and 3-years cumulative overall survival was 93.1%, 86.9%, and 75.1%, respectively. The estimated MST for patients with LD and ED was 29 months (95% CI: 24.0–33.6 months) and 15 months (95% CI: 2.3–19.5 months), respectively.

**TABLE 1:** Characteristics of 118 patients with SCLC.

Characteristic	N	%
Gender		
Male	106	89.8
Female	12	10.2
Age, median (range), year	59 (30–81)	
Performance scales (KPS)		
70	2	1.7
80	19	16.1
90	97	82.2
Weight loss		
Yes	31	26.3
No	87	73.7
Smoking status		
Yes	33	28.0
No	85	72.0
VALG stage			
LD	54	45.8	
ED	64	54.2	
T stage			
T1	16	13.6	
T2	54	45.8	
T3	21	17.8	
T4	27	22.9	
N stage			
N0	5	4.2	
N1	10	8.5	
N2	38	32.2	
N3	65	55.1	
M stage			
M0	55	46.6	
M1	63	53.4	
TNM stage			
Ⅰ	3	2.5	
Ⅱ	7	5.9	
Ⅲ	46	39.1	
Ⅳ	62	52.5	
Initial chemotherapy			
EP	102	86.4	
Non-EP	16	13.6	
Curative thoracic radiotherapy			
Yes	60	50.8	
No	58	49.2	
PCI			
Yes	38	32.2	
No	80	67.8

### Maximum Standardized Uptake Value and Serum Lactate Dehydrogenase in the Primary Tumors

**FIGURE 2:**
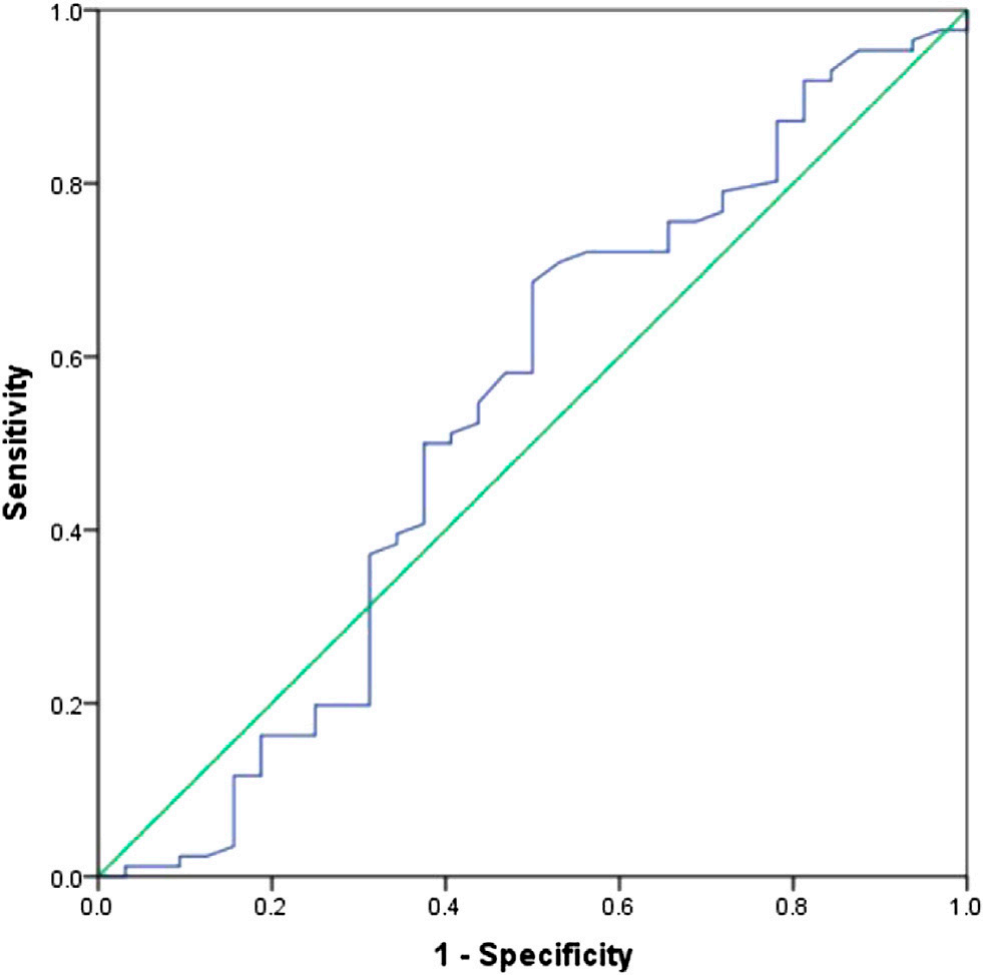
ROC curve of SUV_max_ of primary tumors in 118 patients with SCLC.

The median SUV_max_ in the primary tumors of all patients was 11.85 (6.0–32.8). The ability of SUV_max_ in the primary tumors to predict prognosis was depicted by the ROC curve. The AUC of SUV_max_ was 0.535 (95% CI: 0.407–0.663). The point which showed the maximal sum of sensitivity and specificity was determined to be the cut-off point. As a result, the optimal cut-off value of SUV_max_ was 10.95. Figure 2 shows the ROC curve of SUV_max_.

The median serum LDH in all patients in this study was 226.6 (118.5–1,266.1) U/L. The median serum LDH in patients with LD was 222.8 (118.5–530.3) U/L, with elevated levels in 19 patients (35.2%, 19/54) and normal levels in 35 patients (64.8%, 35/64). The median serum LDH in patients with ED SCLC was 241.2 (132.0–1,266.1) U/L, with elevated levels in 31 patients (48.4%, 31/64) and normal levels in 33 patients (51.6%, 33/64). Serum LDH was significantly different in patients with LD and ED (*p* = 0.003), with patients with LD had lower LDH level.

### Survival Analysis

Univariate analysis of OS for patients with all SCLC determined that M stage, curative thoracic radiotherapy, PCI and the combination of primary tumor SUV_max_ ≤ 10.95 and LDH ≤ 245 U/L were significant predictors, whereas KPS, smoking status, T stage, N stage, SUV_max_, and serum LDH alone, were not significant prognostic factors (Table 2). In multivariate analysis, independent prognostic factors associated with OS included curative thoracic radiotherapy (HR 0.606, *p* = 0.033), PCI (HR 0.325, *p* = 0.000) and the combination of primary tumor SUV_max_ ≤ 10.95 and LDH ≤ 245 U/L (HR 2.417, *p* = 0.005) (Table 3).

**TABLE 2:** Univariate analysis of prognosis in 118 patients with SCLC.

	OS		
	*p *Value	HR	95% CI
KPS	0.077	0.956	0.909–1.005
Smoking status	0.297	0.779	0.488–1.245
T stage	0.851	0.979	0.784–1.222
N stage	0.054	1.290	0.995–1.672
M stage	0.000*	2.252	1.443–3.517
Curative thoracic radiotherapy	0.000*	0.417	0.269–0.645
PCI	0.000*	0.313	0.189–0.517
LDH ≤ 245 U/L	0.676	0.913	0.594–1.401
SUV_max_ ≤ 10.95	0.116	0.688	0.432–1.096
LDH ≤ 245 U/L + SUV_max_ ≤ 10.95	0.032*	0.517	0.284–0.943

* *p* < 0.05.

**TABLE 3:** Multivariate analysis of overall survival in 118 patients with SCLC.

	*p *Value	HR	95% CI
Curative thoracic radiotherapy	0.033*	0.606	0.382–0.961
PCI	0.000*	0.325	0.188–0.563
LDH ≤ 245 U/L + SUV_max_ ≤ 10.95	0.005*	0.414	0.222–0.770

* *p* < 0.05.

Univariate analysis of OS for LD SCLC revealed that KPS, smoking status and PCI and the combination of primary tumor SUV_max_ ≤ 10.95 and LDH ≤ 245 U/L were significant predictors, whereas T stage, N stage, curative thoracic radiotherapy, SUV_max_, and serum LDH alone, were not significant prognostic factors (Table 4). In multivariate analysis, independent prognostic factors associated with OS included smoking status (HR 0.364, *p* = 0.013), PCI (HR 0.180, *p* = 0.000), the combination of primary tumor SUV_max_ ≤ 10.95 and LDH ≤ 245 U/L (HR 0.182, *p* = 0.004) (Table 5).

**TABLE 4:** Univariate analysis of prognosis in 54 patients with LD SCLC.

	*p *Value	HR	95% CI
KPS	0.033*	0.901	0.818–0.991
Smoking status	0.023*	0.425	0.203–0.888
T stage	0.436	0.863	0.597–1.219
N stage	0.646	0.925	0.664–1.288
Curative thoracic radiotherapy	0.235	0.609	0.269–1.381
PCI	0.006*	0.351	0.166–0.745
LDH ≤ 245 U/L	0.407	0.733	0.352–1.527
SUV_max_ ≤ 10.95	0.099	0.519	0.238–1.132
LDH ≤ 245 U/L + SUV_max_ ≤ 10.95	0.018*	0.298	0.110–0.811

* *p* < 0.05.

**TABLE 5:** Multivariate analysis of overall survival in 54 patients with LD SCLC.

	*p *Value	HR	95% CI
Smoking status	0.013*	0.364	0.163–0.810
PCI	0.000*	0.180	0.075–0.433
LDH ≤ 245 U/L + SUV_max_ ≤ 10.95	0.004*	0.182	0.057–0.582

* *p* < 0.05.

Univariate analysis of OS for ED SCLC revealed that N stage and PCI were significant predictors, whereas KPS, smoking status, T stage, curative thoracic radiotherapy, SUV_max_, serum LDH alone, and the combination of primary tumor SUV_max_ ≤ 10.95 and LDH ≤ 245 U/L were not significant prognostic factors (Table 6). In multivariate analysis, independent prognostic factors associated with OS included N stage (HR 2.013, *p* = 0.004), PCI (HR 0.333, *p* = 0.003) (Table 7).

**TABLE 6:** Univariate analysis of overall survival in 64 patients with ED SCLC.

	*p *Value	HR	95% CI
KPS	0.878	0.995	0.934–1.060
Smoking status	0.646	1.159	0.617–2.178
T stage	0.675	1.065	0.793–1.431
N stage	0.010*	1.916	1.171–3.134
Curative thoracic radiotherapy	0.056	0.542	0.289–1.017
PCI	0.007*	0.383	0.1896–0.774
LDH ≤ 245 U/L	0.321	1.310	0.768–2.232
SUV_max_ ≤ 10.95	0.980	1.007	0.562–1.807
LDH ≤ 245 U/L + SUV_max_ ≤ 10.95	0.650	1.192	0.559–2.538

* *p* < 0.05.

**TABLE 7:** Multivariate analysis of overall survival in 64 patients with ED SCLC.

	*p *Value	HR	95% CI
N stage	0.004*	2.013	1.251–3.239
PCI	0.003*	0.333	0.162–0.684

* *p* < 0.05.

### Subgroup Analysis of Maximum Standardized Uptake Value Combined With Serum Lactate Dehydrogenase

Base on the ROC curve, the optimal cut-off value of SUV_max_ was 10.95. The AUC was 0.535 (95% CI: 0.407–0.663). The patients were divided into four groups according to SUV_max_ and serum LDH. Group A for primary tumor SUV_max_ > 10.95 and LDH > 245 U/L, Group B for primary tumor SUV_max_ > 10.95 and LDH ≤ 245 U/L, Group C for primary tumor SUV_max_ ≤ 10.95 and LDH > 245 U/L, and Group D for primary tumor SUV_max_ ≤ 10.95 and LDH ≤ 245 U/L.

Among the patients with LD, there were 12 patients in group A, with an MST of 28 months (95% CI: 12.92–43.08); 19 patients in group B, with an MST of 28 months (95% CI: 19.33–36.67); seven patients in group C, with an MST of 27 months (95% CI: 11.79–42.21); and 16 patients in group D, with an MST of 72 months (95% CI: 26.00–118.0). There was no significant difference of MST among the different groups (*p* = 0.057). Among the patients with ED, there were 22 patients in group A, with an MST of 21 months (95% CI: 11.16–30.84); 22 patients in group B, with an MST of 14 months (95% CI: 9.40–18.60); nine patients in group C, with an MST of 14 months (95% CI: 0–33.00); and 11 patients in group D, with an MST of 11 months (95% CI: 8.68–13.32). There was no significant difference of MST among the four groups (*p* = 0.742).

**FIGURE 3:**
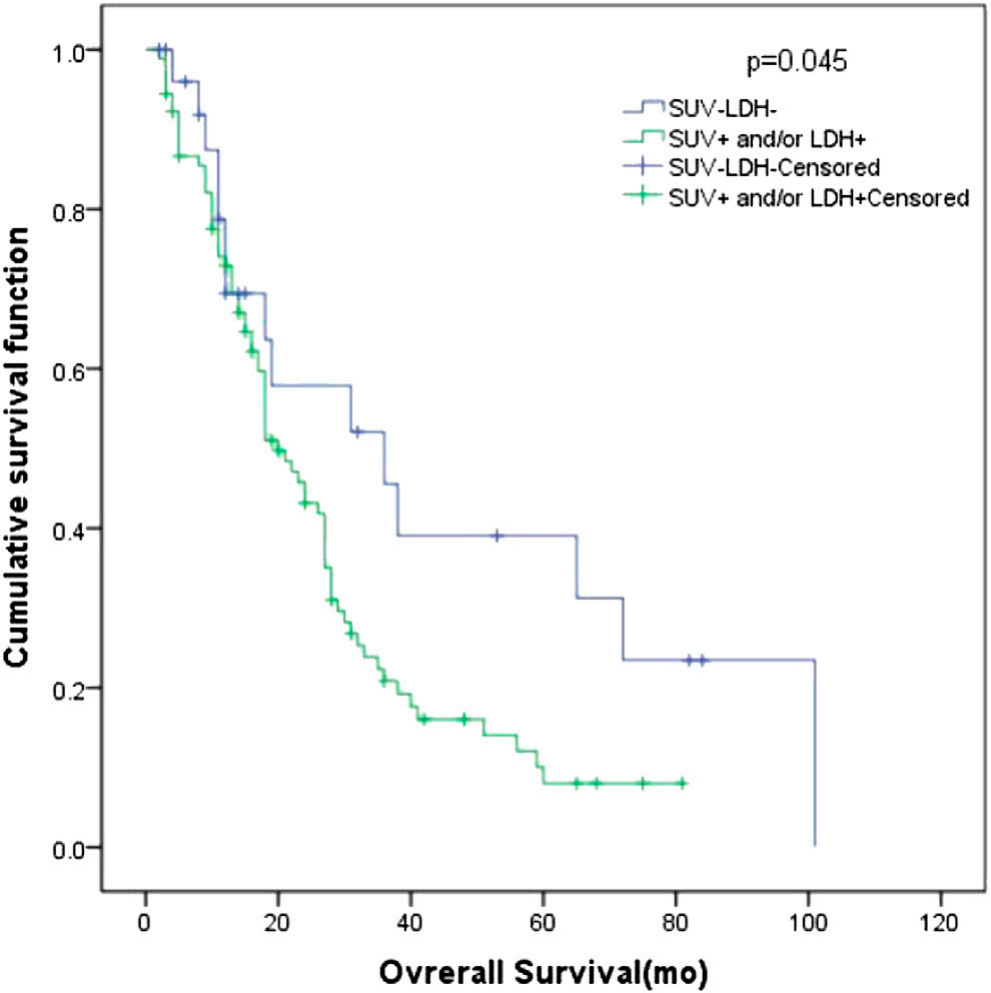
Survival curves of SUV_max_ of different primary tumor and LDH in 118 patients with SCLC.

In all patients, there were 27 patients in group D (primary tumor SUV_max_ ≤ 10.95 and LDH ≤ 245 U/L), with an MST of 36 months (95% CI: 12.98–59.02). There were 91 patients with SUV_max_ > 10.95 and/or LDH > 245 U/L (group A + B + C), and their MST was 20 months (95% CI: 15.47–24.53). The difference of MST between these two groups in the study was statistically significant (*p* = 0.045) (Figure 3).

**FIGURE 4:**
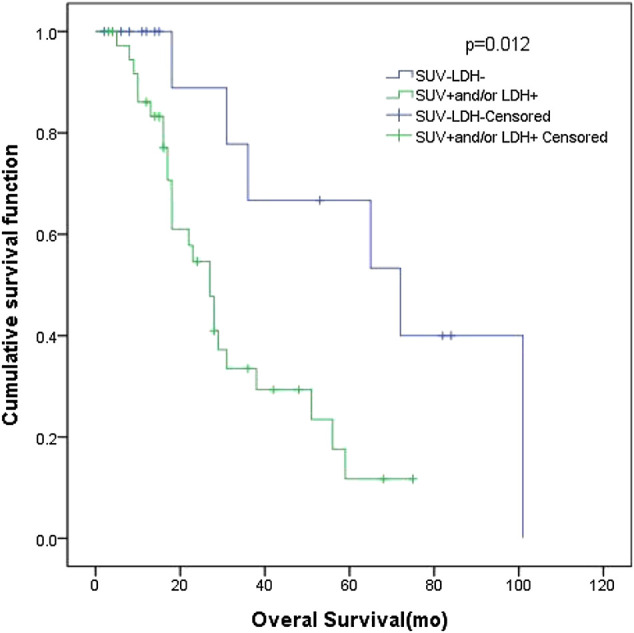
Survival curves of SUV_max_ of different primary tumor and LDH in 54 LD SCLC patients.

In patients with LD SCLC, there were 16 patients in group D (primary tumor SUV_max_ ≤ 10.95 and LDH ≤ 245 U/L), with an MST of 72 months (95% CI: 26.00–118.0). There were 38 patients with SUV_max_ > 10.95 and/or LDH > 245 U/L (group A + B + C), and their MST was 27 months (95% CI: 20.80–33.21). The difference of MST between these two groups was statistically significant (*p* = 0.012) (Figure 4).

**FIGURE 5:**
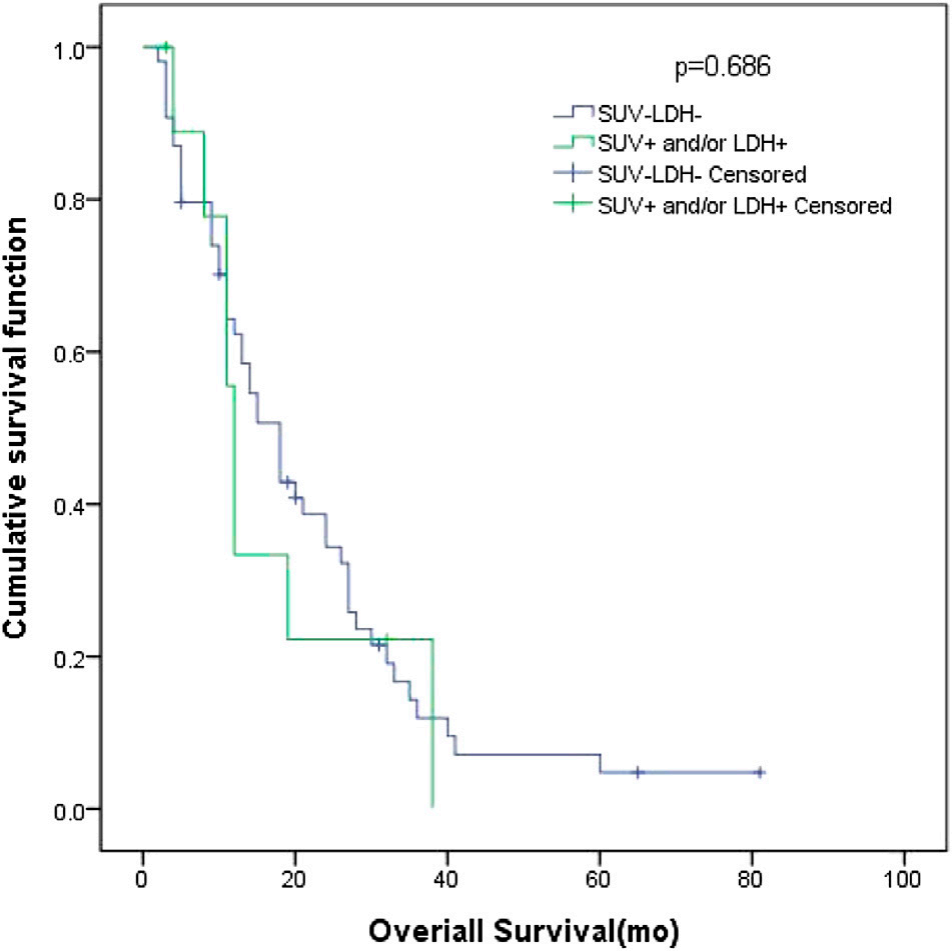
Survival curves of SUV_max_ of different primary tumor and LDH in 64 ED SCLC patients.

In patients with ED SCLC, there were 10 patients in group D (primary tumor SUV_max_ ≤ 10.95 and LDH ≤ 245 U/L), with an MST of 18 months (95% CI: 13.69–22.32). There were 54 patients with SUV_max_ > 10.95 and/or LDH > 245 U/L (group A + B + C), and their MST was 12 months (95% CI: 10.61–13.39). The difference of MST between these two groups was not statistically significant (*p* = 0.686) (Figure 5).

## Discussion

A number of factors, including tumor stage, performance status, weight loss, tumor burden and elevated tumor biomarkers, have been indicated to affect survival prognosis in SCLC (Kalemkerian and Schneider, 2017). Conventionally, tumor stage is determined by anatomical images, which are used to indicate the tumor size and border. However, the anatomical image-based tumor volume does not represent the tumor burden. Functional imaging can provide metabolic information on malignant tissues and thus more accurately reflects tumor burden. SUV is a semiquantitative index of ^18^F-FDG uptake in tumors. SUV can reflect the metabolic information of tumors. Previous studies indicated that the SUV_max_ of primary tumors is helpful for predicting prognosis of patients with cancers (Warburg, 1956; Romero-Garcia et al., 2016).

The application of ^18^F-FDG PET/CT in SCLC remains controversial. Some studies have shown that ^18^F-FDG PET/CT imaging can provide prognostic information. (van der Leest et al., 2012) analyzed the correlation of primary tumor SUV_max_ and survival data, including OS and progression-free survival time (PFS) in SCLC. Compared with stage IV patients, stage I-III patients had lower SUV_max_ in the primary tumors. In this study, SUV_max_ alone did not distinguish difference of OS or PFS. However, among patients with stage IV and receiving chemotherapy, patients with higher SUV_max_ had worse prognosis than those with lower SUV_max_. The relationship between SUV_max_ and prognosis appears to be more complicated in SCLC than in NSCLC.

In a study by Pandit et al. (2003), a high SUV_max_ was associated with poor survival with significance. ^18^F-FDG PET could be important for tumor staging and follow-up, because there was a significant negative correlation between SUV_max_ and survival. The overall survival in PET-negative patients was significantly better than that in PET-positive cases. (Lee et al., 2009) also found that tumor metabolic activity as assessed by ^18^F-FDG PET is a significant prognostic factor. It could identify high risk of death in both LD and ED SCLC subgroups of patients. Oh et al. (2012) found that WBMTV (whole body metabolism tumor volume), instead of SUV_max_ or LDH, was an independent predictor of progression and death in 106 patients with SCLC who underwent ^18^F-FDG PET/CT before treatment. Incorporation of WBMTV with TNM staging can provide a more detailed prediction of prognosis than WBMTV with conventional staging as well as tumor staging alone. In the subgroup analysis of LD SCLC, Kwon et al. (2016) found that the highest SUV_max_ was a prognostic factor for PFS with marginal significance (HR: 1.078, *p* = 0.053). After adjusting for age, sex, performance status, tumor stage, and treatment modality, patients with higher SUV_max_ (>11) were characterized by a significantly shorter median OS (*p* < 0.001) and PFS (*p* = 0.002) compared with patients with lower SUV_max_. The high SUV_max_ was an independent prognostic factor for survival in LD-SCLC patients and might be a possible imaging biomarker for risk stratification in LD-SCLC.

In contrast, Ong’s study (Ong et al., 2016) suggests that pretreatment PET scans, even with the use of advanced metrics, do not have independent prognostic value for outcomes in LD patients after chemoradiotherapy (CRT). They found that SUV_max_, SUV_mean_, MTV, and total lesion glycolysis of the primary tumor were not significantly associated with OS, LRF, and DFS in univariate analysis. MTV was significantly associated with DFS (*p* = 0.024) in univariate but not multivariate analysis in 120 patients with LD who received platinum-based chemotherapy and a median radiation dose of 45 Gy. Kim’s study (Kim and Chang, 2015) showed no significant differences in OS and PFS between high and low SUV_max_ groups in baseline PET/CT in a survival analysis of 82 patients with SCLC.

We performed this retrospective analysis with the following hypotheses. PET/CT reflects the integrated results of glucose transport and the first key enzyme in the process of glycolysis. PET/CT can be recorded with images and has quantitative indexes such as SUV. Serum LDH is the total amount of enzyme released into the blood by cells, reflects the product of glycolysis, and can be tested in peripheral blood. Thus, PET/CT and serum LDH represent the input and output of glycolysis, respectively. Different values of SUV_max_ and serum LDH could represent different prognoses in patients with SCLC.

This retrospective study shows that SUV_max_ of the primary tumor combined with serum LDH is an important independent prognostic factor for overall survival and progression-free survival. This parameter is a good predictor of survival in patients with SCLC. The prognosis of LD SCLC patients with primary tumor SUV_max_ ≤ 10.95 and a normal level of serum LDH is better than those of patients with primary tumor SUV_max_ > 10.95 and/or serum LDH, with a longer overall survival time (MST of 72 and 27 months, respectively). The prognosis of all SCLC patients with primary tumor SUV_max_ ≤ 10.95 and a normal level of serum LDH was better than that of patients with primary tumor SUV_max_ > 10.95 and/or serum LDH, with longer overall survival time (MST of 36 and 20 months, respectively). These results indicate that the prognosis is better in the cases of low SUV_max_ and LDH, i.e., when both the input and output of glycolysis are low.

There are some limitations in this study. First, it is a retrospective study with a small sample size. Second, two different PET/CT scanners were used in this study. However, our regular quality control ensured scanning consistency. In addition, volume metabolic parameters (such as MTV and TLG) were not included in the study. Because nearly half of the cases in this study were ED SCLC, it is difficult to achieve a Volume Of Interest (VOI) including all lesions of the whole body excluding physiological or benign avid lesions automatically. Moreover, manual drawing is more likely to be influenced by the operator. Therefore, volume metabolic parameters were not used in this study. Further studies with large sample size and multiple centers and prospective studies are needed.

## Conclusion

Combination of ^18^F-FDG PET/CT and serum LDH could be a prognostic factor of overall survival of patients with SCLC. The prognosis of patients with limited disease of SCLC who have low SUV_max_ of the primary tumor and normal serum LDH was better than those with high SUV_max_ and/or high serum LDH.

## Data Availability Statement

The raw data supporting the conclusions of this article will be made available by the authors, without undue reservation.

## Ethics Statement

The studies involving human participants were reviewed and approved by IRB Sun Yat-Sen University Cancer Center. Written informed consent for participation was not required for this study in accordance with the national legislation and the institutional requirements.

## Author Contributions

XL: Conceptualization, methodology, software, data curation, visualization, investigation, writing-original draft preparation, and writing-editing. ZX, YH, and XZ: Data curation and visualization. WF: Conceptualization, supervision, validation, and writing-reviewing.

## Funding

This work was supported by the Natural Science Foundation of Guangdong Province, China, No. 2018A030310239.

## Conflict of Interest

The authors declare that the research was conducted in the absence of any commercial or financial relationships that could be construed as a potential conflict of interest.
